# On the Treatment of Dysentery

**Published:** 1812-07

**Authors:** 


### *To the Editors of the Medical and Physical Journal.*

GENTLEMEN,

THE difference of opinion existing amongst medical practitioners, in regard to the treatment of disorders of the most frequent occurrence, will probably still continue a subject of regret to the profession; to prove the truth of the remark, we have merely to refer to recent publications on gout, apoplexy, scarlatina, chorea sancti viti, &c. &c. The above remark was more particularly elicited by reading that passage in Scott's Medical Sketches, in your Journal

Journal for April, where he reprobates the use of calomel in dysentery, as adding "stimulus to stimulus," when given as a purgative, and speaks highly of the early exhibition of opium; his reasoning, in my opinion, is inconclusive, and the treatment he so strenuously advises does not accord with that recommended by many eminent practitioners. Zimmerman, in his treatise on dysentery, trusted the cure chiefly to aperient medicines, and disapproves of administering opium till the bowels had been previously evacuated; observing, that, although the ill consequences of a contrary practice may not be immediately felt, yet subsequent complaints will evince the impropriety of such treatment, which he considered as absolutely dangerous. Dr. Heberden, in his Commentarii de Morborum Hist. et Curat. makes a similar observation; he recommends the neutral salts, which had the advantage of affording present ease, and removing, by effectual evacuations, the cause of the disorder. He only permitted opium to be given in clysters, at the decline of the complaint; if earlier exhibited, he considered it as prejudicial, which was the opinion of Huxham,* and the majority of medical writers since his time. Dr. Pemberton writes decidedly in favor of purgatives; he says, in the treatment it is necessary to attend to the *constant* degree of pain, as well as to the nature of the discharge per anum. If the pain is violent and unremitting, he advises venesection; and observes, "the patient perpetually complains of a load in the bowels; and, unless this sensation is removed, by removing the cause, that is, by the completest evacuation of the scybala, the purgatives applied have not performed their office." Besides the Ol. Ricini and Magnes. Sulphas, he advises a pill of Hydrarg. Submur. gr. v. every three or four days; as the patient expresses the fulness of the bowels, the calomel is directed, as milder purgatives would not exert sufficient power to dislodge the hardened fœces.

The mercurial plan of treatment in dysentery, by W. Fergusson, in the second volume of the Medico-Chirurgical Transactions, consisted in giving half a grain of calomel with one of ipecacuan, every four hours, till the mouth was affected: he considered opium to be hurtful, and even dangerous, the temporary ease it afforded from the tormina was generally succeeded by worse symptoms; when the excessively acrid discharge that came from the bowels was

* "Si astringentia et opiata præpropere dantur mox gravissime accedunt tormina, stomachi ægritudo, singultus, aphthæ, tandemque intestinorum sphacelus, quem cito mors excipit."

examined, examined, it was easily to be seen that it could not be locked up in the intestines with impunity. Dr. Gourlay found benefit from exhibiting six, eight, or ten, grains of calomel, repeated at intervals, and determined in their length of time by the previous effect of its action, and other circumstances of the patient; and, in regard to astringents or opiates, he seldom found occasion for them, unless where the free evacuation of the intestines was neglected in the first instance. Calomel proved the best remedy in the beginning for unloading the bowels and relieving uneasy symptoms, and in the latter stage the best and softest anodyne. Dr. Gray is an advocate for the employment of mercury by friction, in the proportion of ʒj. of the Ung. Hydrarg. Fort. two, three, or four, times a-day, so as to induce ptyalism; this plan was very successful, but he indulges no conjectures as to the modus operandi of the remedy. The proximate cause has long been subject of argument: one imagines a constriction of the colon; another, that the bowels are not the primary cause of the complaint, but merely secondary; another imputes the mischief to hardened scybala; another to the *peculiar* morbid inflammation of the intestines; *specific* contagion; marsh miasma. It would be endless to enumerate the proximate causes of dysentery assigned by various authors; truly might Virgil exclaim, "Felix, qui potuit rerum cognoscere *causas*."

Mr. Scott imagines the remote cause of this formidable complaint to be "a peculiar state of the intestinal canal, produced by the unwholesome stimulus of certain ingesta for a given time;" if so, purgatives must be the most appropriate remedy to evacuate these *certain* ingesta; but neither clysters, opium, nor venesection, could exert sufficient effect, unaided by cathartics. I do not dispute the truth of what Mr. S. advances, as to his medical friend in Portugal, who uses opium so freely; but, from my own trifling experience, and on the authority of the respectable writers I have quoted, I am inclined to condemn the *early* exhibition of opiates, as highly prejudicial; and, till farther trial shall evince their perfect safety and utility in preference to purgatives, shall continue an advocate for the latter treatment, which is admirable in theory, and will, in my opinion, be found no less so in practice. The successful mercurial treatment of Fergusson and Dr. Gray may possibly throw some light on the proximate cause of this disease, of which, to be candid, we are hitherto unacquainted with. We must lament the obscurity which still exists in the pathology of many disorders, but this, at the same time, should animate our exertions to surmount difficulties, by impartial inquiry and

 minute minute investigation. Unfortunately, invective but too frequently supercedes liberal argument, and speculative theories are blazoned unsupported by experience; in proof of the former, we have only to read partial *criticisms* on the works of judicious authors, and of the latter we have recently beheld, in the writings of a man* of the highest abilities, a mind lost in the boundless regions of imagination.

I am, Gentlemen,
Your most obedient Servant,
*Hythe, April* 28, 1812. T. E. B.

* On pourait rapeller encore les *merveilles* de la médecine pneumato-chimique, si vantée par Beddoes, et rejectée à cette heure, par l'auteur même qui l'avait prônée."—*Rapport sur le Cow-pox, par A. Aubert, D. M.*

